# Regulation of EBNA1 protein stability and DNA replication activity by PLOD1 lysine hydroxylase

**DOI:** 10.1371/journal.ppat.1010478

**Published:** 2023-06-01

**Authors:** Jayaraju Dheekollu, Andreas Wiedmer, Samantha S. Soldan, Leonardo Josué Castro- Muñoz, Christopher Chen, Hsin-Yao Tang, David W. Speicher, Paul M. Lieberman

**Affiliations:** The Wistar Institute, Philadelphia, Pennsylvania, United States of America; Harvard University, UNITED STATES

## Abstract

Epstein-Barr virus (EBV) is a ubiquitous human γ-herpesvirus that is causally associated with various malignancies and autoimmune disease. Epstein-Barr Nuclear Antigen 1 (EBNA1) is the viral-encoded DNA binding protein required for viral episome maintenance and DNA replication during latent infection in proliferating cells. EBNA1 is known to be a highly stable protein, but the mechanisms regulating protein stability and how this may be linked to EBNA1 function is not fully understood. Proteomic analysis of EBNA1 revealed interaction with Procollagen Lysine-2 Oxoglutarate 5 Dioxygenase (PLOD) family of proteins. Depletion of PLOD1 by shRNA or inhibition with small molecule inhibitors 2,-2’ dipyridyl resulted in the loss of EBNA1 protein levels, along with a selective growth inhibition of EBV-positive lymphoid cells. PLOD1 depletion also caused a loss of EBV episomes from latently infected cells and inhibited *oriP*-dependent DNA replication. Mass spectrometry identified EBNA1 peptides with lysine hydroxylation at K460 or K461. Mutation of K460, but not K461 abrogates EBNA1-driven DNA replication of *oriP*, but did not significantly affect EBNA1 DNA binding. Mutations in both K460 and K461 perturbed interactions with PLOD1, as well as decreased EBNA1 protein stability. These findings suggest that PLOD1 is a novel interaction partner of EBNA1 that regulates EBNA1 protein stability and function in viral plasmid replication, episome maintenance and host cell survival.

## Author summary

EBNA1 is essential for EBV latent infection and pathogenesis. We found that PLOD family of lysine hydroxylases interacts with EBNA1 and that PLOD1 contributes to EBNA1 protein stability and function in viral persistence during viral latent infection. We identified lysine hydroxylation at EBNA1 K460 or K461, and mutations in K460 compromised EBNA1 function in *oriP*-dependent DNA replication. Identification of PLOD1 regulation of EBNA1 protein stability and function may provide new opportunities to target EBNA1 for degradation in EBV associated disease.

## Introduction

Epstein-Barr Virus (EBV) is a human γ-herpesvirus that establishes life-long latent infection in over 90% of the adult population world-wide [1,2]. EBV latent infection is a causative factor for several cancers, including Burkitt lymphoma (BL), nasopharyngeal carcinoma (NPC), and post-transplant lymphoproliferative diseases (PTLD) [3–5]. EBV is also associated with several autoimmune diseases, especially multiple sclerosis (MS) where viral proteins have been implicated as the molecular mimic and trigger for auto-reactive antibodies and T-cells [6,7].

Epstein-Barr Nuclear Antigen 1 (EBNA1) is the viral-encoded sequence-specific DNA-binding protein that binds to tandem repeats in the viral origin of plasmid replication (*oriP*) and is required for viral episome maintenance and plasmid replication during latent infection in proliferating cells [8,9]. EBNA1 can also modulate transcription of viral and host genes, and interacts with host proteins that are implicated in viral oncogenesis, such as USP7 and CK2 [10–12]. EBNA1 is predominantly localized to the nucleus of infected cells and is the most consistently detected protein in EBV-associated tumors. EBNA1 is also known to have a relatively long half-life (~20 hrs) in B-cells [13]. Factors that control EBNA1 protein stability are not fully understood. EBNA1 glycine-alanine repeats have been shown to control translation to prevent the production of defective ribosomal products (DRiPs) [14–17]. EBNA1 also contains two SUMO-interacting motifs that regulate interactions with ubiquitin modifying enzymes STUB1 and USP7, and also contributes to EBNA1 DNA binding and episome maintenance function [18]. However, it remains unclear how these various factors regulate EBNA1 protein stability and function.

The Procollagen-Lysine,2-Oxoglutarate 5-Dioxygenases (PLODs) are required for the post-translational modification that allows collagen cross-links and maturation of extracellular matrix (reviewed in [19]). PLOD1, 2 and 3 have different roles in collagen modification including a glycosylase activity unique to PLOD3. PLODs are expressed at different levels in different tissue types. While inherited mutations in PLODs cause connective tissue disorders, such as Ehlers-Danlos syndrome [20], upregulation of PLODs have been associated with several cancers, including gastric cancers and hepatocellular carcinomas [19,21–25]. A recent study has found that PLOD1 and 3 can interact with EBNA1 in transfected AGS gastric carcinoma cells with preferential binding to EBNA1 isoforms found in epithelial cancers [26]. Here, we further advance these pioneering studies to show that EBNA1 can interact with all three PLODs and that depletion of PLOD1, or small molecule inhibition of PLOD enzymatic activity leads to a loss of EBNA1 protein stability and function in *oriP*-dependent DNA replication and episome maintenance. We also provide evidence that EBNA1 is subject to lysine hydroxylation at residues that regulate interaction with PLOD1 and *oriP*-dependent DNA replication.

## Results

### EBNA1 proteomics identifies interaction with PLOD family of lysine hydroxylase

**Fig 1:**
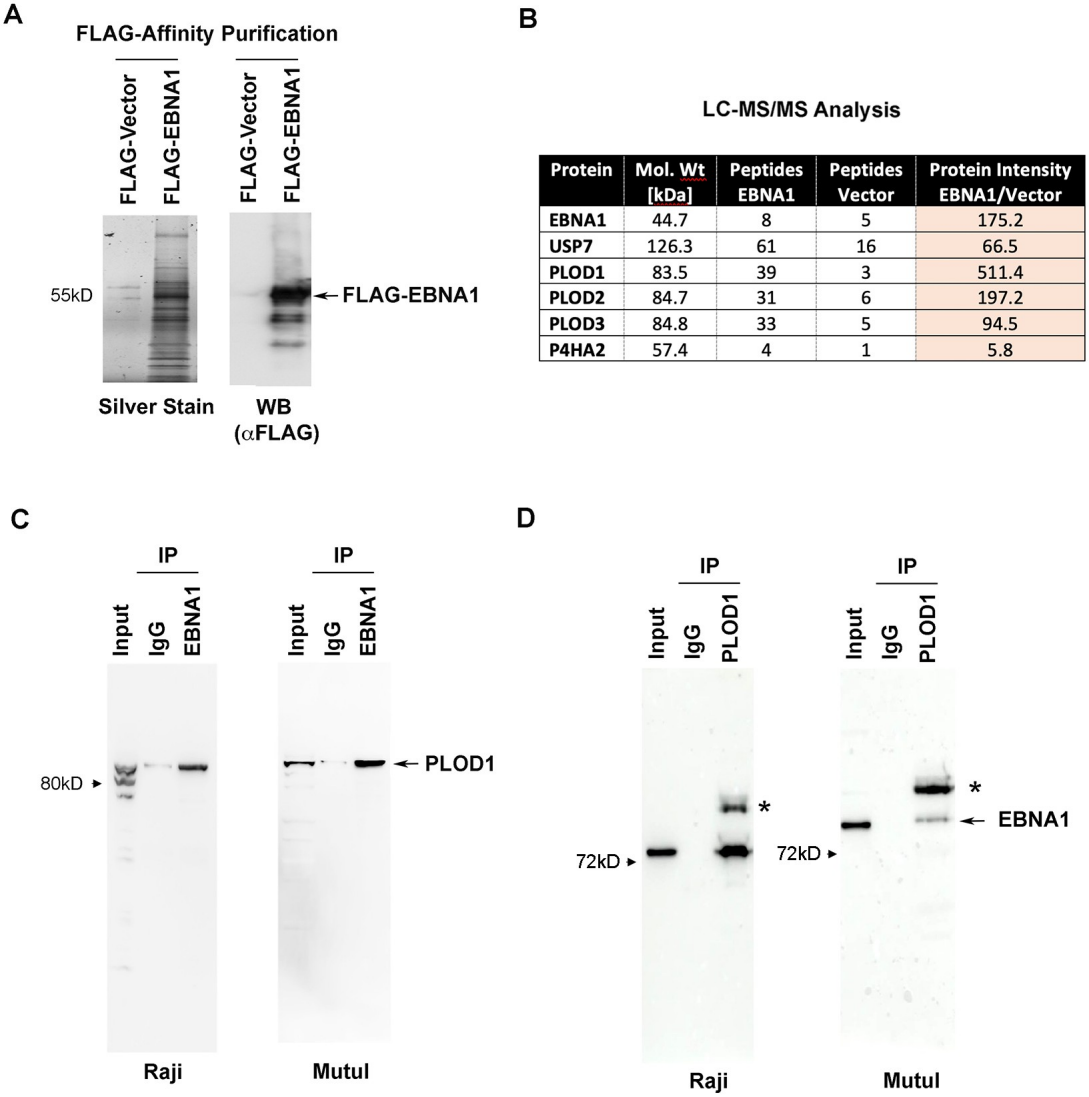
Identification of PLODs as EBNA1 interaction partners. **A)** FLAG-affinity purified proteins from HEK293T cells with stable expression of FLAG-EBNA1 or FLAG-Vector from *oriP* plasmids were analyzed by silver stain (left) or FLAG Western blot (WB, right). **B)** LC-MS/MS analysis of FLAG-EBNA1 associated proteins highlighting numbers of peptides identified for USP7, PLOD1, PLOD2, PLOD3, and P4HA2. **C)** Immunoprecipitation (IP) with EBNA1 or IgG control antibody from Raji (left) or MutuI (right) total cell extracts probed by Western blot with antibody to PLOD1. **D)** Same as in C, except reciprocal IP with PLOD1 antibody probed with antibody to EBNA1. * indicates an unknown slower mobility species reactive to EBNA1 antibody.

We have previously reported an LC-MS/MS proteomic analysis of EBNA1 [27]. For these studies, FLAG-EBNA1 was expressed from stable *oriP*-containing episomes to enrich for cellular proteins that bound to EBNA1 in the functional context of *oriP*. We report here the identification of PLOD1, 2, and 3 as proteins highly enriched in FLAG-EBNA1 fraction relative to the FLAG-vector control (Fig 1A and 1B). We also identified USP7, which has been well-characterized for its interaction with EBNA1 [11], and P4HA2, a proline hydroxylase related to PLODs (Fig 1B). RNA analysis of PLODs revealed that two isoforms of PLOD1 (A and B) were expressed at higher levels than PLOD2 or PLOD3 in EBV+ B-cell lines (S1 Fig). We therefore focused our efforts on characterization of PLOD1 with EBNA1 in these B-cell lines. Immunoprecipitation (IP) with endogenous EBNA1 in Raji and Mutu I BL cell lines revealed selective enrichment of PLOD1 relative to IgG control (Fig 1C). Similarly, reverse IP with PLOD1 in Raji and Mutu I cells revealed selective enrichment of EBNA1 relative to IgG control (Fig 1D). We also observed a slower mobility species (*) reactive to EBNA1 antibody in the PLOD1 IP, but were unable to confirm this species as a post-translational modification of EBNA1.

### Inhibitor of PLOD1 leads to loss of EBNA1

**Fig 2:**
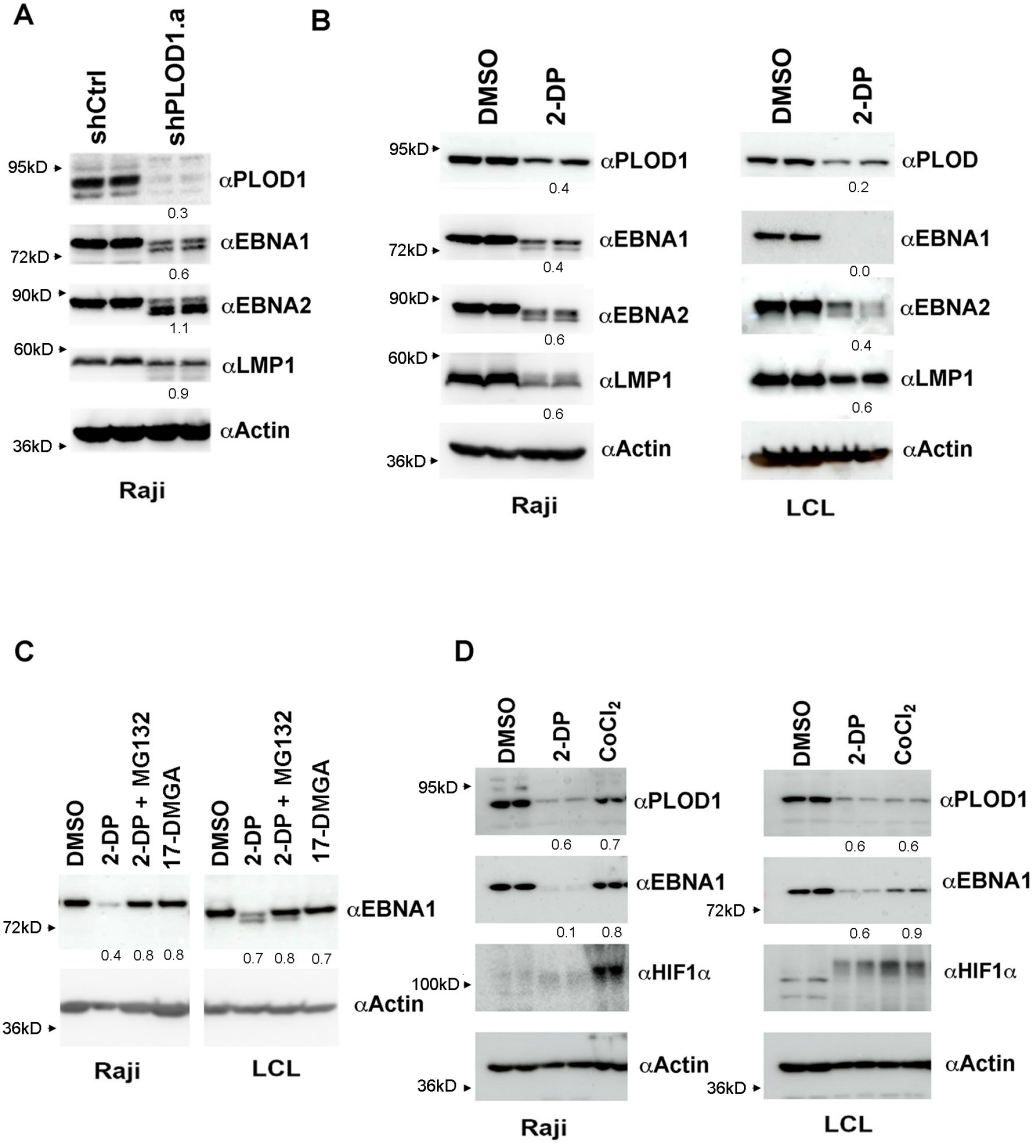
PLOD1 shRNA depletion and small molecule inhibition with 2-DP destabilize EBNA1 protein. **A)** Raji cells transduced with lentivirus shControl (shCtrl) or shPLOD1 (pool a) were assayed by Western blot for PLOD1, EBNA1, EBNA2, LMP1, or Actin at 7 days post-transduction. Each lane is a biological replicate. **B)** Raji (left) or LCL (right) treated with DMSO or 2,’2-dipyridil (2-DP, 200 μM) were assayed by Western blot for PLOD1, EBNA1, EBNA2, LMP1, or Actin. **C)** Raji (left) or LCL (right) were treated with DMSO, 2-DP (200 μM), 2-DP+MG132 (10 μM), or 17-DMGA (1 μM) for 48 hrs and assayed by Western blot for EBNA1 (top) or Actin (bottom). **D)** Raji (left) or LCL (right) were treated with DMSO, 2-DP (200 μM), or CoCl_2_ (100 μM) for 48 hrs followed by Western blot for PLOD1, EBNA1, HIF1α, or Actin.

To investigate the potential effects of PLOD1 on EBNA1 protein expression, we first tested lentiviruses expressing shRNAs targeting PLOD1. We found that efficient depletion of PLOD1 required combinations of pooled shRNAs targeting PLOD1. Using these pooled shRNAs, we observed a significant loss of expression of PLOD1 in Raji BL cells (Fig 2A, **top panel**), as well as in Mutu I and LCLs (S2A Fig). In the same knock-down of PLOD1, we observed a reduction in EBNA1 protein levels (Figs 2A and S2A), and in some conditions a faster migrating species (Fig 2A). We observed a similar change in EBNA2 and LMP1, while cellular actin was not affected (Fig 2A). shRNA depletion of PLOD1 did not lead to a detectable increase in EBV lytic proteins ZTA or EA-D in Raji cells, but resulted in a modest increase in ZTA expression in LCLs (S2A Fig). Using siRNA as an alternative to shRNA, we found that siRNA pool targeting PLOD1 in 293-EBV Bacmid containing cells led to a reduction in both PLOD1 and EBNA1 protein levels, with no significant increase in lytic activator ZTA (S2B Fig).

To determine if these effects of PLOD1 protein depletion correlated with loss of PLOD1 enzymatic activity, we next used a small molecule inhibitor of PLOD1. Bipyridine (also known as 2,2 dipyridil and referred to here as 2-DP) has been reported to be a selective inhibitor of PLOD1 [28]. We found that treatment of Raji and LCLs with 2-DP (100 μM) led to a loss of EBNA1 and EBNA2 in both cell types, with less of an effect on LMP1 or cellular actin (Fig 2B), thus phenocopying shRNA depletion of PLOD1. To determine if the loss of EBNA1 protein levels were partly due to proteosome degradation, we assayed the effects of 2-DP in combination with proteosome inhibitor MG132 (Fig 2C). We found that MG132 stabilized EBNA1 protein in the presence of 2-DP, suggesting that 2-DP leads to proteosomal degradation of EBNA1. EBNA1 protein can be destabilized by other small molecules, such as the HSP90 inhibitor 17-DMGA [29]. We found that 17-DMGA did not lead to the loss of EBNA1 as did 2-DP under these conditions. Since 2-DP has the potential to chelate iron and induce hypoxia stress response, we compared the effects of 2-DP to treatment of CoCl_2_ a known inducer of hypoxic stress response through stabilization of HIF1A (Fig 2D). We found that 2-DP led to a loss of PLOD1 and EBNA1 in both Raji and LCL, and stabilized HIF1A modestly in LCLs only. In contrast, CoCl_2_ stabilized HIF1A in both Raji and LCL, and reduced PLOD1 and EBNA1 in LCL, but had only weak effects on PLOD1 and EBNA1 in Raji cells. These findings suggest that 2-DP may inhibit PLOD1 through mechanisms distinct from HSP90 inhibition or hypoxia stress response, although there may be some cell-type dependencies with these pathways.

### Inhibition of PLOD1 selectively block EBV^+^ B cell survival

**Fig 3:**
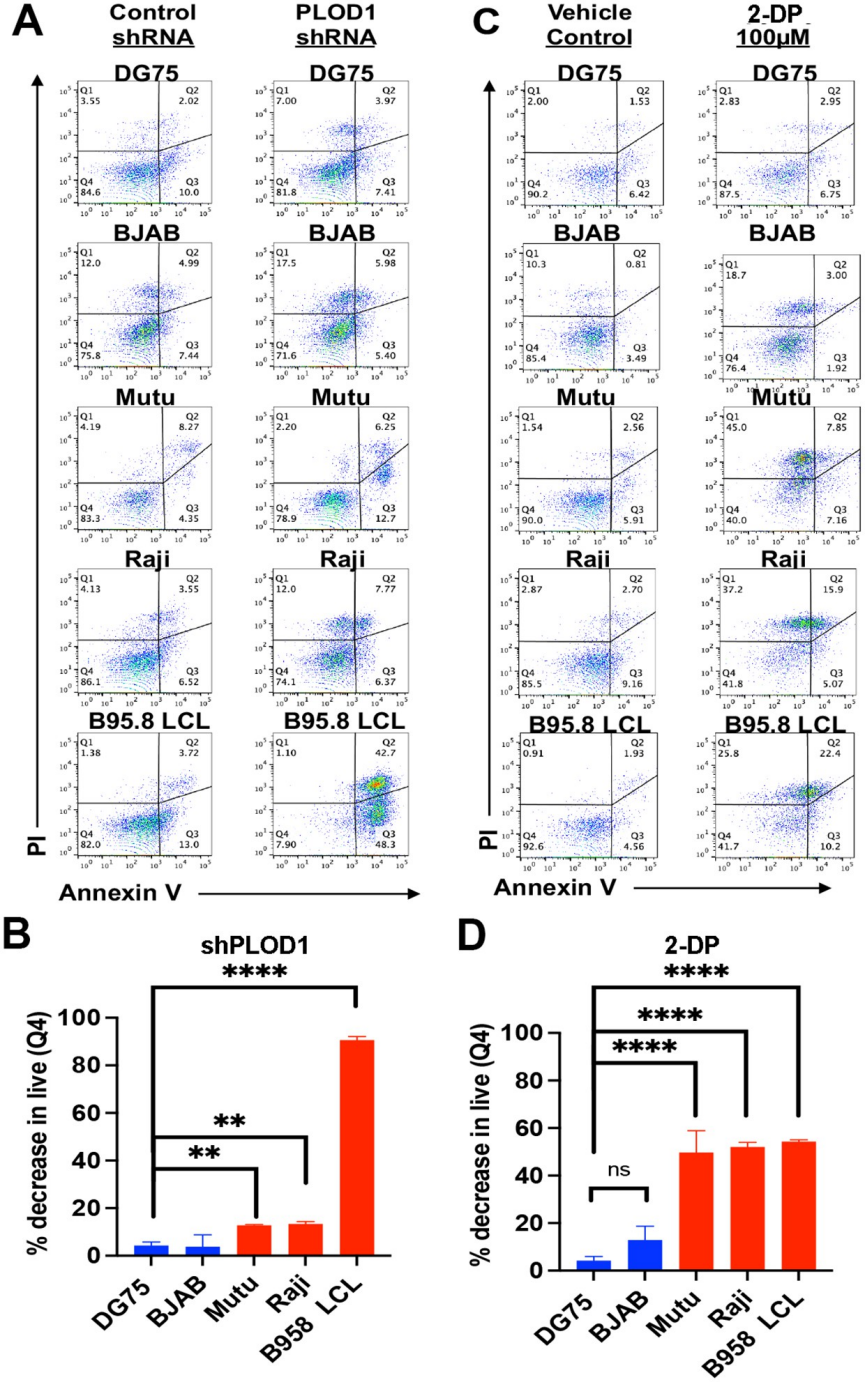
Selective inhibition of EBV-positive cells to PLOD1 depletion or inhibition by 2-DP. **A)** Flow cytometry analysis of cell viability using propidium iodide (PI, y-axis) and Annexin V (x-axis) for DG75, BJAB, MutuI, Raji, B95.8LCL transduced with control shRNA or PLOD1 shRNA for 5 days. **B)** Quantification of the % decrease in live cells in treatments described in panel A. EBV positive cells (red bars), EBV negative cells (blue bars). **C)** Same as in panel A, except treatment with DMSO or 2-DP (200 μM). **D)** Quantification of the % decrease in live cells in treatments described in panel C. P-values determined by ordinary one-way ANOVA and Dunnett’s multiple comparison test: **** p<0.0001, ** p<0.01, * p = 0.0108.

We next tested the effects of PLOD1 shRNA depletion and inhibition by 2-DP on EBV-dependent cell growth and survival (Fig 3). We compared EBV positive cells B-cell lines (Raji and MutuI BL and B95.8 transformed LCLs) with EBV negative B-lymphoma cell lines (BJAB and DG75). Cells were treated with 100 μM 2-DP for 2 days or with lentivirus transduction of shPLOD1 for 5 days followed by FACS profiling for propidium iodide (PI) and annexin V staining. We found that both shPLOD1 and 2-DP induced a significant decrease in the percentage of proliferating/live cells (Q4) for EBV-positive MutuI, Raji, and LCL relative to EBV-negative BJAB and DG75. LCLs were particularly sensitive to shPLOD1-mediated depletion (Fig 3B). These findings suggest that EBV positive lymphoid cells are more sensitive than EBV negative lymphoid cells to loss of PLOD1 protein and its enzymatic activity.

### PLOD1 contributes to EBV episome maintenance in latently infected B-lymphocytes

**Fig 4:**
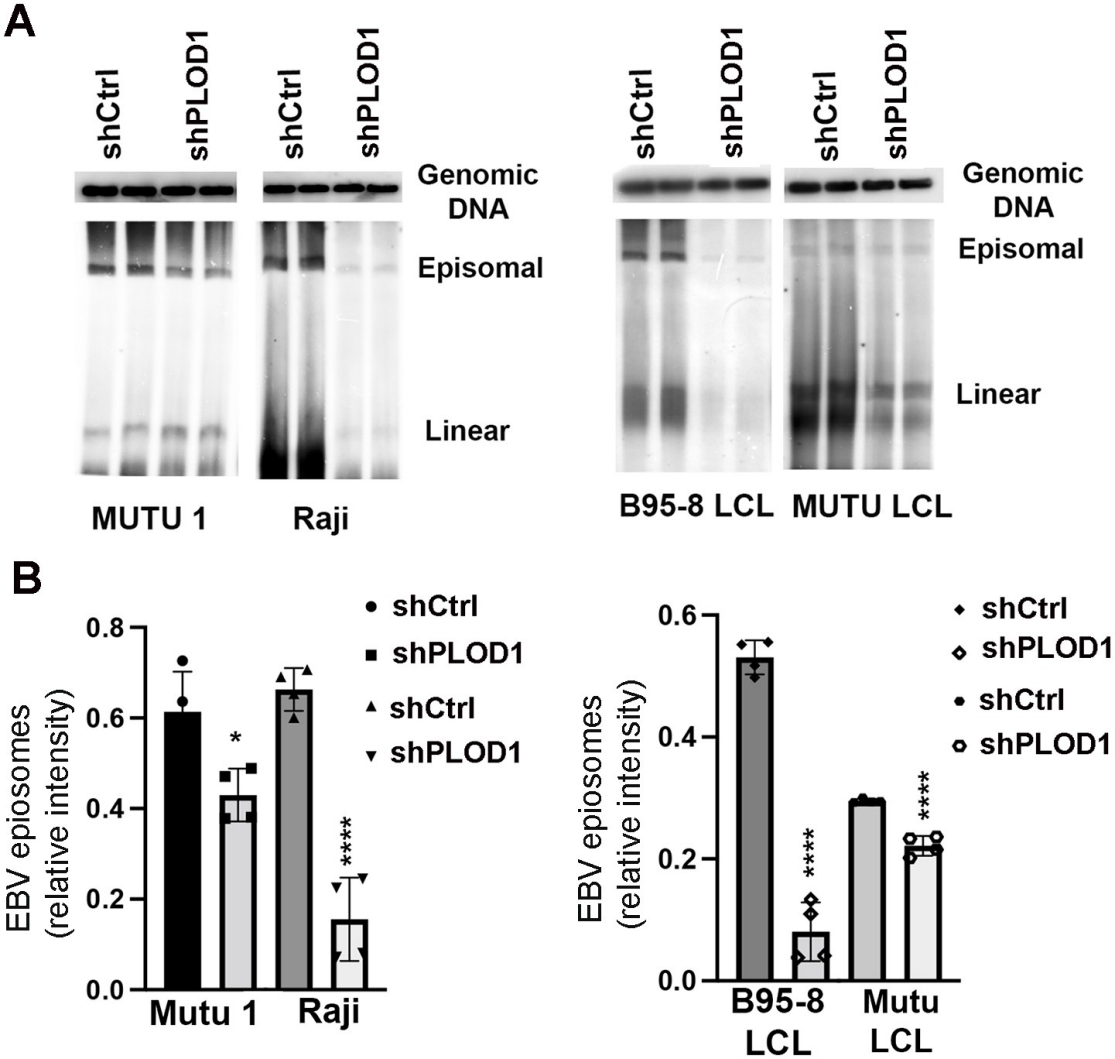
PLOD depletion causes loss of EBV episomes. **A)** PFGE analysis of MutuI, Raji, or B95-8 or Mutu LCLs transduced with lentivirus expressing shCtrl or shPLOD1 for 7 days and then analyzed by Southern blot for Genomic DNA (top) or EBV BamHI W repeat (lower panel) indicating viral episomes or linear genomes. Numbers under each panel represents relative average protein abundance normalized to actin and input. **B)** Quantification EBV episomes from PFGE shown in panel C. P-values determined by ordinary one-way ANOVA and Dunnett’s multiple comparison test: **** p<0.0001, * p = 0.0108.

We next assayed the effects of PLOD1 depletion on the maintenance of EBV episomes in two different BL (Mutu I and Raji) and LCL (transformed with B95-8 or Mutu virus) cell lines (Fig 4). PFGE analysis revealed that shPLOD1 depletion caused a significant loss of EBV episomal DNA in each cell type (Fig 4A and 4B). The efficiency of shPLOD1 depletion was measured by RT-qPCR and Western blot for each cell type (S3 Fig) and the positions of EBV episomes and linear DNA were aligned with PFGE molecular weight markers (S4 Fig). These findings suggests that PLOD1 depletion leads to a loss of EBV episomes from latently infected LCLs and BL cell lines.

### PLOD1 contributes to EBNA1-dependent DNA replication

**Fig 5:**
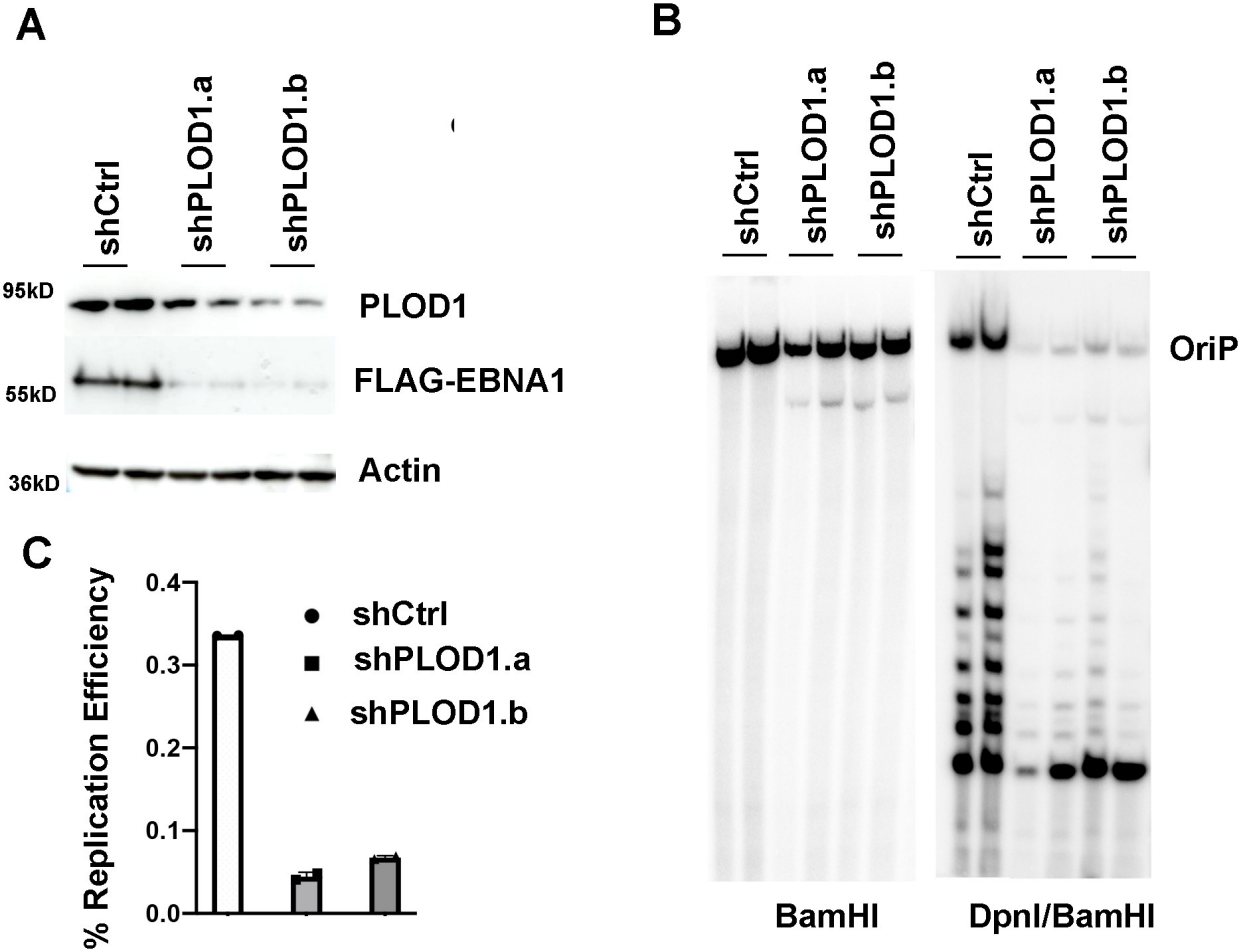
PLOD depletion inhibits EBNA1-dependent DNA replication of *oriP* plasmids. **A)** HEK293T cells transfected with *oriP* plasmids expressing FLAG-EBNA1 were transfected with expression vectors for shCtrl, shPLOD1.a, shPLOD1.b and assayed by Western blot for PLOD1 (top), FLAG-EBNA1 (middle) or Actin (bottom) at 3 days post-transfection. **B)** *oriP*-plasmid replication for cells treated as in panel A assayed by Southern blot after BamHI digest (left) or DpnI/BamHI (right). Undigested linear oriP plasmid DNA is indicated. Each lane represents a biological replicate. **C)** Quantification of % replicated *oriP* DNA for experiments shown in panel B.

To determine if PLOD1 affected EBNA1 DNA replication function, we assayed transient plasmid replication in HEK293 cells transfected with *oriP*-containing plasmids that also expressed FLAG-EBNA1. We assayed two different pools of PLOD1 shRNAs (shPLOD1.a and shPLOD1.b) for their ability to efficiently deplete PLOD1. While both shPLOD1.a and shPLOD1.b led to a modest reduction in PLOD1 protein at this time point, the depletion on FLAG-EBNA1 expression was substantial (Fig 5A). We then assayed the effect of shPLOD1 on EBNA1-dependent DNA replication. We found that both shPLOD1.a and shPLOD1.b reduced *oriP*-dependent DNA replication as measured by DpnI resistance assay and Southern blot detection of *oriP*-containing plasmid DNA (Fig 5B and 5C). These findings further support the role for PLOD1 in the stabilization of EBNA1 protein levels, and its functional importance for *oriP*-dependent DNA replication.

### Lysine hydroxylation of EBNA1

**Fig 6:**
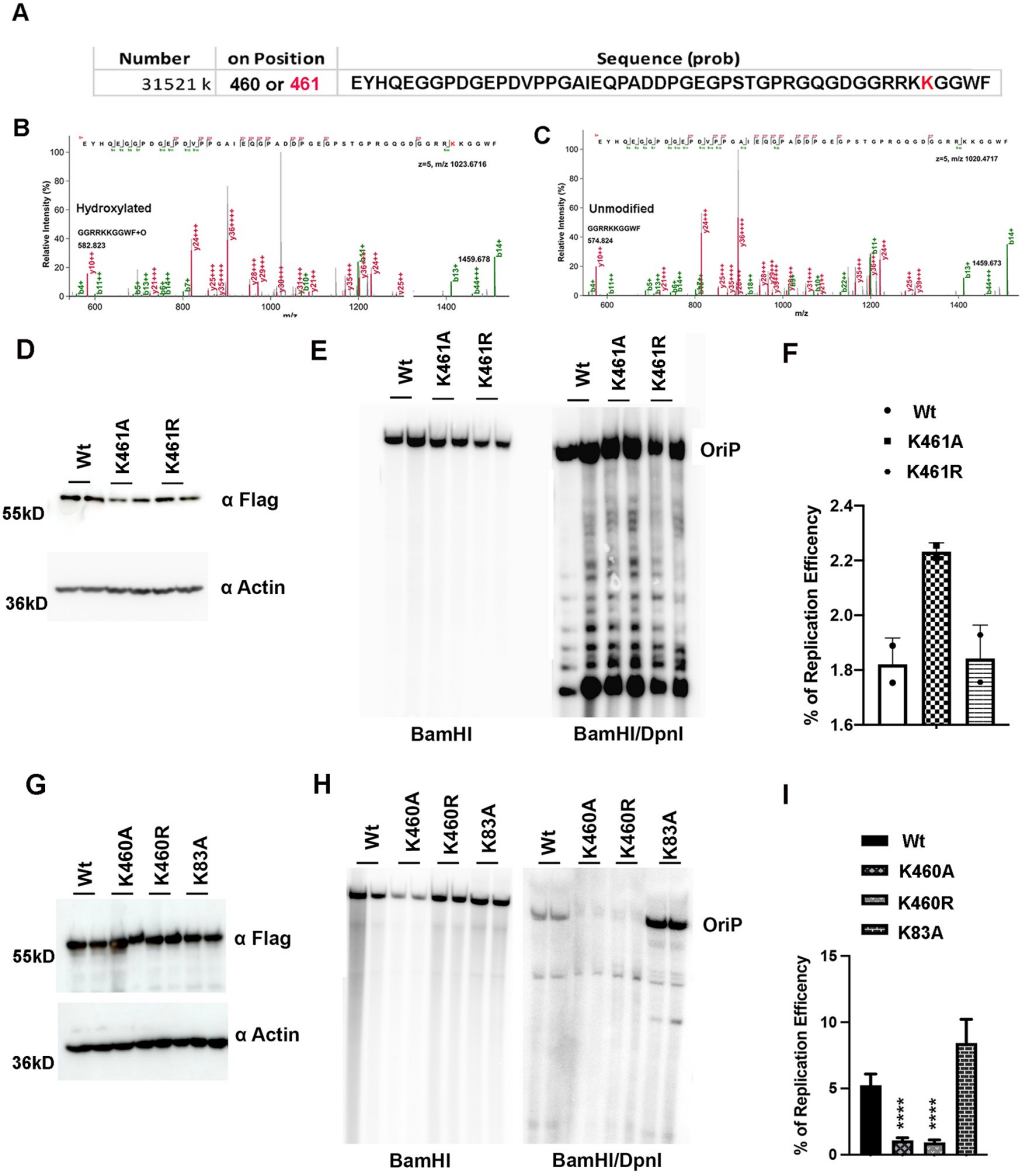
Evidence for lysine hydroxylation of EBNA1. **A)** Mass spectrometry (MS) of EBNA1 peptide with a mass/charge shift consistent with lysine hydroxylation. Consensus PLOD1 substrate recognition site at K461 highlighted in red. **B)** MS/MS spectrum of EBNA1 peptide with hydroxylation. **C)** MS/MS spectrum of the unmodified EBNA1 peptide. **D)** Western blot analysis of EBNA1 Wt, K461A, or K461R expressed in HEK293T cells. **E)** Southern blot analysis of *oriP*-dependent DNA replication for EBNA1 Wt, K461A, or K461R. **F)** Quantification of % *oriP* DNA replication shown in panel E. **G)** Western blot analysis of EBNA1 Wt, K460A, or K460R expressed in HEK293T cells. **H)** Southern blot analysis of *oriP*-dependent DNA replication for EBNA1 Wt, K460A, or K460R. **I)** Quantification of % *oriP* DNA replication shown in panel H. P-values determined by ordinary one-way ANOVA and Dunnett’s multiple comparison test: **** p<0.0001.

To investigate the possibility that EBNA1 may be subject to post-translational modification through lysine hydroxylation, we performed LC-MS/MS analysis of immunoprecipitated EBNA1. We identified one peptide with a mass/charge (m/z) shift consistent with a single lysine hydroxylation (Fig 6A–6C). The EBNA1 peptide aa 416–465 had two potential lysine residues that could be hydroxylated, K460 and K461. PLOD1 typically hydroxylates lysines that precede glycine, which is found for K461 [19]. We therefore first tested whether mutations in K461 impacted EBNA1 function in *oriP*-dependent DNA replication (Fig 6D-F). We found that K461A had a modest stimulatory effect, while K461R had no significant effect on *oriP* replication (Fig 6D-F). We next asked whether mutations in the neighboring K460 had any effects on *oriP*-DNA replication (Fig 6G-I). We also included a mutation in K83A, which also has a PLOD1 consensus recognition site, and has been previously implicated in the PLOD1 interaction with the EBNA1 N-terminus [26]. All EBNA1 mutants were expressed at similar levels in HEK293T cells (Fig 6G). We found that K83A had a modest enhancement of *oriP* replication, while both K460A and K460R reduced *oriP* replication >5-fold (Fig 6H and 6I). We also found that mutations in both K460A and K461A bound to *oriP* similar to wild-type EBNA1 as measured by ChIP assay, suggesting that these effects are not due to the disruption of EBNA1-DNA binding (S5 and S6 Figs). We next tested whether any of these mutations affect EBNA1 interaction with PLOD1 in co-immunoprecipitation assay (S7 Fig). We found that interaction of EBNA1 with PLOD1 was slightly enhanced by K460A, but reduced by K461A or combined mutation of K460A/K461A. PLOD1 interaction with EBNA1 was not affected by K83A under our experimental conditions which are in contrast to previous studies [26], but may be explained by different experimental conditions, such as the presence of *oriP* DNA substrate in our experimental design. Finally, we assayed the effects of K460/K461 mutations on EBNA1 protein stability (S8B Fig). We found that K460/K461R, but not K460/K461A had a modest reduction in EBNA1 protein stability (S8 Fig). Taken together, these findings indicate that EBNA1 can be hydroxylated on either K460 or K461 and that mutation of K460 disrupt DNA replication function. These results also indicate that PLOD1 interaction and EBNA1 protein stability are partly dependent on K460 and K461.

## Discussion

**Fig 7:**
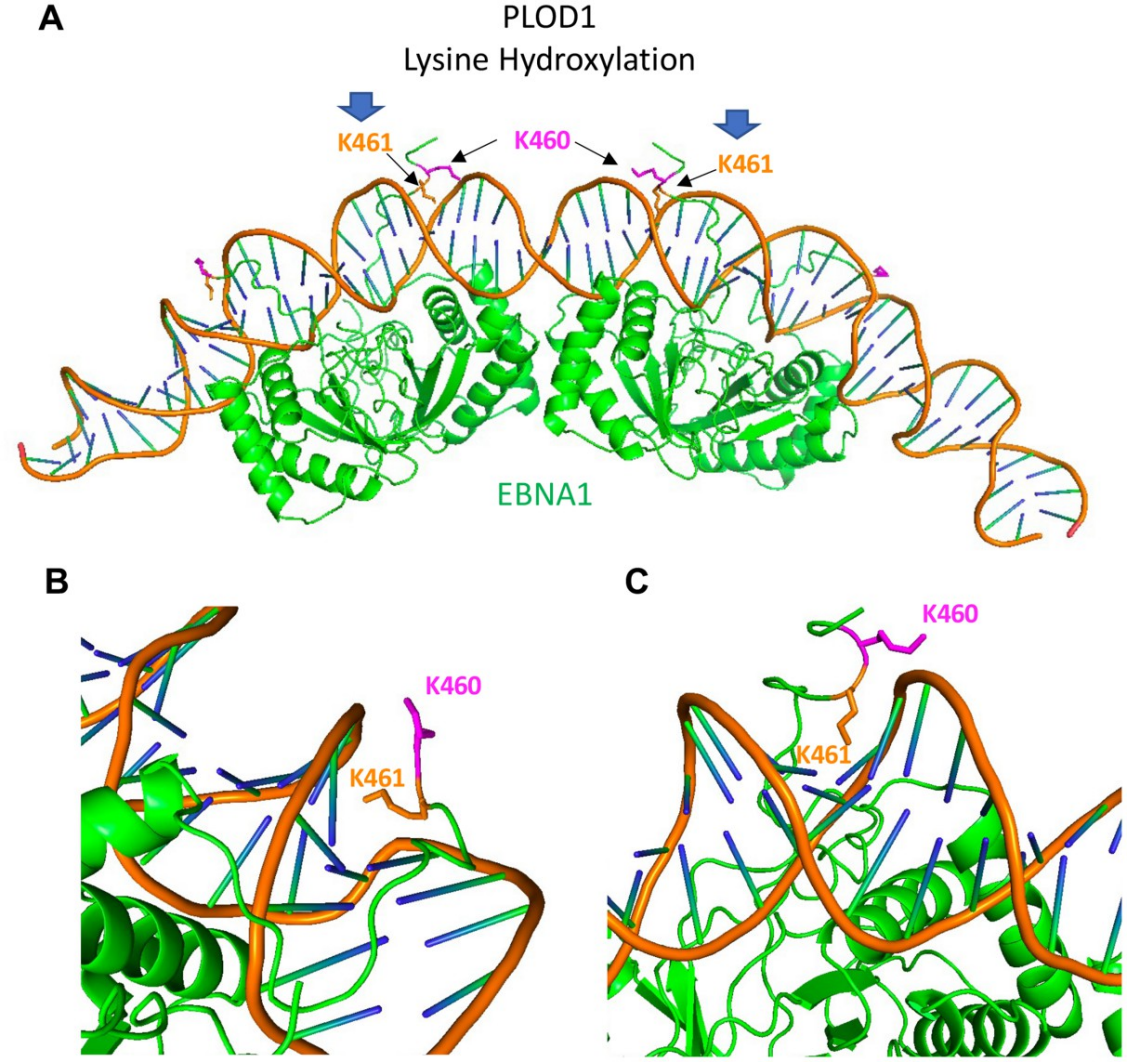
Model of CRYO-EM structure showing variable orientations of EBNA1 K460 and K461 bound to *oriP* DS element. **A)** The CRYO-EM structure of 4 EBNA1 monomers bound to the half-DS (PDB 71UT) showing K460 (magenta) and K461(orange). **B and C)** Two different view points of EBNA1 monomers with different conformations of K460 and K461.

Protein stability and function are known to be co-regulated for many processes, including transcription and replication [30,31]. Herein, we describe an EBNA1 interaction partner, PLOD1, that contributes to EBNA1 protein stability and essential functions in episome maintenance and DNA replication. We identified PLODs 1, 2, and 3 as EBNA1-associated proteins by LC-MS/MS and validated the interaction with PLOD1 antibody and coIP experiments in transfected 293HEK, and with native proteins in latently infected LCLs and BL cells. We found that shRNA and siRNA depletion of PLOD1 led to a loss of EBNA1 protein levels in various cell types tested. A small molecule inhibitor of PLOD1, namely 2-DP, phenocopied PLOD1 depletion by reducing the levels of EBNA1 protein. 2-DP and PLOD1 depletion led to loss of cell viability in an EBV-dependent manner. PLOD1 depletion also led to a loss of EBV episomes in latently infected B-cells, as well as a reduction in *oriP*-dependent DNA replication in HEK293 cells. Finally, we used mass spectrometry to identify EBNA1 peptides with mass/charge shifts consistent with lysine hydroxylation at K460 or K461. While mutations at K461 had only small effects on EBNA1 replication activity, mutations of K460 strongly attenuated EBNA1 replication function. Mutations at K460 increased PLOD1 binding, while mutations at K461 decreased PLOD1 binding, and a double K460R/461R decreased EBNA1 protein stability. The positions of K460 and K461 are in close proximity to the DNA minor grove recognized by the N-terminal extension of the DNA binding domain (Fig 7) [32]. The position of these amino acids adopt variable conformation in different monomers in the DS and FR as determined by CRYO-EM structure and modeling [32]. We propose that hydroxylation of K461 (or K460) regulates the conformation or accessibility of K460 to modulate replication function and protein stability.

PLOD1 and PLOD3 have previously been reported to interact with EBNA1 [26]. In this earlier study, PLOD1 was found to bind preferentially to EBNA1 with a polymorphism (T85A) frequently associated with NPC and EBVaGC and to modulate EBNA1 transcriptional activity [26]. PLOD1 interaction with EBNA1 was found to be dependent on K83, a lysine residue in the N-terminal domain that also conforms to a consensus substrate for PLOD1 hydroxylation. Consistent with this previous study, we did not observe any defect in DNA replication associated with K83A mutation (Fig 6H). However, we found that PLOD1 could still bind to EBNA1 with K83A mutation (S8 Fig). This discrepancy with previous published study may be due to technical differences, including the presence of *oriP* DNA in our IP conditions. It was proposed that EBNA1 interaction with PLOD1 may sequester PLOD1 away from other substrates, such as procollagen, to drive tumorigenesis [26]. Our findings provide new information on the role of PLODs in the regulation of EBNA1 protein stabilization and function in DNA replication and episome maintenance. Our data suggests that PLODs directly affect EBNA1 stabilization and function through post-translational modification at residues immediately adjacent to the DNA binding domain of EBNA1 (Fig 7).

PLODs have been implicated in several human disease, including cancers [19]. Although PLODs are best characterized for lysine hydroxylation of pro-collagen during the maturation of extracellular matrix [33], our data suggests that they can bind and modify other substrates, such as EBNA1. PLODs utilize iron and alpha-ketoglutarate as cofactors, so it is likely that metabolic conditions regulate PLOD activity. The PLOD inhibitor 2-DP may compete with iron, and iron chelators have been implicated in regulating EBV latent to lytic switch [34]. Thus, PLOD regulation of EBNA1 protein stability and function may reflect a mechanism for coordinating EBV latency with cellular metabolism. Protein stabilization can be integrally linked to many functions, including transcriptional activation [30] and replication origin function [35]. Hydroxylation of proline is another well-known modification that regulates protein stability, such as for HIF1A in the hypoxic response [36]. Interestingly, the prolyl hydroxylase P4HA2 was also identified in the LC-MS/MS of EBNA1 interacting proteins suggesting that EBNA1 may be subject to additional modifications in response to metabolic changes. Modifiers of metabolism and small molecule inhibitors of PLOD1, such as 2-DP, may provide new strategies to degrade and inactivate EBNA1 to treat EBV-associated disease.

There are a number of limitations to our study. PLOD1 shRNA may have off-target effects that contribute to the observed effects on EBNA1 protein stability and function. We used pools of shRNA for lentivirus transduction, as well as siRNA pool, and found consistent reduction in EBNA1 when PLOD1 was efficiently depleted. It is possible that PLOD1 depletion may lead to other indirect effects, which could account for some of the effects on EBNA1 and cell viability. We also observed a decrease in EBNA2 and LMP1 after shPLOD1 or 2-DP treatment, suggesting that PLODs can regulate other EBV latency proteins, although this could be indirect effects on EBV episome maintenance. We were unable to distinguish hydroxylation on K460 or K461, and only K460 mutations significantly affected EBNA1 replication. Mutations in K460 and K461 had only minor effects on EBNA1 stability and did not phenocopy PLOD1 knock-down, suggesting that PLOD1 may have additional activities regulating EBNA1 protein expression levels. While mutation of K460 ablated EBNA1 DNA replication function, this activity may be partly independent of PLOD1. Future studies will be required to dissect the specific role of EBNA1 lysine hydroxylation on protein stability and DNA replication, and how these activities are mechanistically linked to PLOD1.

## Materials and methods

### Cells, plasmids, and shRNAs

EBV-positive Burkitt’s lymphoma cells MutuI, Raji, MutuI virus-derived lymphoblastoid cell line (LCL) and, B95-8 LCL were grown in RPMI 1640 medium (Gibco BRL) containing 15% fetal bovine serum and antibiotics penicillin and streptomycin (50 U/ml). HEK 293T cells were culture in Dulbecco’s modified Eagle’s medium (DMEM) with 10% fetal bovine serum and antibiotics. All the cells were cultured at 37°C and 5% CO_2_ environment. Mammalian expression vector for Flag-EBNA1 contained B95-8 EBNA1 lacking the GA repeats (aa 101–324) under the control of CMV-3XFLAG promoter in a plasmid derived from pREP10 (Clontech) containing, *oriP*, GFP, and hygromycin resistance [27]. Small hairpin RNAs (shRNAs) for PLOD1 (shPLOD1), and the control (shControl) were obtained from the Sigma/TRC (The RNAi Consortium) collection of targeted shRNA plasmid library (TRC no. 62248, 62249, 62259, 62251and 62252). Lentivirus particles were generated in 293T-derived packaging cell lines as pools of either 62248+62249 (shPLOD1.a) or 62259+62251 (shPLOD1.b). shRNA targeting sequences are GCCGACTATTGACATCCACAT (62248), GCCCTATATTTCAAACATCTA (62249), CCCAGAAACACATGCGACTTT (62259), and CTCAAGTTTGAAATGGGCCAT (62251). shControl was generated in pLKO.1 vector with target sequence 5′-TTATCGCGCATATCACGCG-3′. siRNA to PLOD1 was purchased as specific ON-TARGETplus siRNA Smartpool against PLOD1 (Horizon Discovery, Inc) or non-targeting control siRNA. The targeting sequences for the Smartpool for PLOD1 are: cuggacgacucacgcauua; gagaacgugccgacuauug; agagggagcagaucaauau; cuucgucgaucccuaauug.

### Drug treatments

Raji and LCLs (2 × 10^5^ cells/ mL) were treated with vehicle control (DMSO; 0.016%, vol/vol) or 2-DP (200 μM) or 17-DMAG (1 μM) or CoCl_2_ (100 μM) for 48 hr. Cells were harvested and the Western blots were performed. For MG132 studies cells were treated with either vehicle control (DMSO; 0.016%, vol/vol) or 2-DP (200 μM) for 24 hr followed by adding MG132 to a final concentration of 10 μM and continue the treatment for another 24 hr. EBNA1 stability was measured by transfecting 293HEK cells FLAG-EBNA1 WT or mutants followed by treatment with cycloheximide (Sigma Aldrich, Sant Louis, MO, USA) 75 μg/ml at 72 hrs post-transfection and then assaying FLAG-EBNA1 by Western at 0, 12, and 24 hrs post-cyclohexamide treatment.

### Site-directed mutagenesis

Primers (IDT) were designed to generate the point mutations (K461A and K461R) in CMV Flag-EBNA1 containing *oriP* and hygromycin resistance plasmid (N2624). A two-stage PCR protocol for site-directed mutagenesis was adapted from Stratagene [27]. Following DpnI digestion and heat inactivation, PCR products were transformed into DH5α cells. Purified plasmids from colonies were sequenced to confirm the mutation.

### Western blots

The PVDF membranes were blotted with the following antibodies: anti-β-actin-peroxidase (Catalog NO. A3854; Sigma-Aldrich), anti-EBNA1 mouse monoclonal antibody (Catalog NO. sc-81581; Scbt), and anti-Flag M2-peroxidase (horseradish peroxidase [HRP]) (Sigma-Aldrich, cat no. A8592), anti-PLOD1 rabbit polyclonal (Catalog NO. HPA049137; Thermo), anti-PLOD1 rabbit polyclonal (Catalog NO.LS-C482920 and NO.LS-C409466; LSBio), anti-EBNA2 rat polyclonal (Catalog NO. 50175912; Fisher), anti- LMP1 mouse monoclonal (Catalog NO. M0897; Dako), anti-EBNA1 rabbit polyclonal antibodies (custom prepared at Pocono Rabbit Farm), and imaging on a Amersham Imager 680.

### Chromatin immunoprecipitation (ChIP)

ChIP assays were performed as previously described [37]. Briefly, 293T (~1 x 10^6^ cells) were plated in 10 cm dishes. 24 h later cells were transfected with Lipofectamine 2000 (12 μl, Invitrogen) and 4 μg *oriP* plasmids expressing either FLAG-B95-8 EBNA 1or lysine mutation. Cells were split after 48 h, and then harvested at 72 h post transfection for ChIP assay. TaqMan qPCR performed using primers and probe designed by Themo-Fisher at *oriP*). Antibodies used were as follows: anti-IgG mouse monoclonal (Santa Cruz Biotechnology), anti-Flag antibody or anti-FLAG resin (Catalog NO. M8823; Sigma-Aldrich).

### Plasmid replication assays

Plasmid DNA replication assays have been described previously [27,38]. Briefly, 293T (~1 x 10^6^ cells) were plated in 10 cm dishes. 24 h later cells were transfected with Lipofectamine 2000 (12 μl, Invitrogen) and 4 μg *oriP* plasmids expressing either FLAG-B95-8 EBNA 1, with shPLOD1 or shControl plasmids. Cells were split after 48 h, and then harvested at 72 h post transfection for both episomal DNA and protein. Episomal DNA was extracted by Hirt Lysis [39]. The DNA pellets were dissolved in 150 μl of 10 mM Tris HCl, 1 mM EDTA buffer (pH 7.6) and 15 μl was subjected to restriction digestion with BamHI alone and 135 μl was subjected to BamHI and DpnI digestion overnight at 37° C. DNA was extracted with phenol: chloroform (1:1), precipitated, and electrophoresed on a 0.9% agarose gel and transferred to a nylon membrane (PerkinElmer) for Southern blotting. Blots were visualized and quantified using a Typhoon 9410.

### shRNA-mediated knockdown of PLOD1

EBV-positive cells were infected by spin infection with pLKO.1 vector-based lentivirus expressing shRNA targeting PLOD1 or shRNA control. Lentiviruses were produced by cotransfection with envelope and packaging vectors pMD2.G and pSPAX2 in 293T cells. shPLOD1 lentivirus were produced with combination of 2 targeting plasmids, expressing either GCCGACTATTGACATCCACAT (62248), GCCCTATATTTCAAACATCTA (62249), for shPLOD1.a or CCCAGAAACACATGCGACTTT (62259), CTCAAGTTTGAAATGGGCCAT (62251) for PLOD1.b. MutuI, Raji, or LCL cells were infected with lentiviruses carrying pLKO.1-puro vectors by spin-infection at 450 g for 90 minutes at room temperature. The cell pellets were resuspended and incubated in fresh RPMI medium, then treated with 2.5 μg/ml puromycin at 48 hrs after the infection. The RPMI medium with 2.5 μg/ml puromycin was replaced every 2 to 3 days. The cells were collected after 7 days of puromycin selection, then subject to following assays.

For siRNA depletion, 293-EBV WT bacmid cells maintained in DMEM with 10 μg/ml streptomycin, 10 U/ml penicillin, and 150 μg/mL of hygromycin B at 37°C in 5% CO_2_. Cells were transfected using with either a nonspecific siRNA or specific ON-TARGETplus siRNA against PLOD1 in the DMEM media using Dharmfect reagent without 10 μg/ml streptomycin, 10 U/ml penicillin. After 24 hours, cells were re-transfected, collected after 72 hours, and prepared for Western blot analysis.

### EBV episome maintenance by pulsed-field electrophoresis

MutuI, Raji, and LCLs were infected with lentivirus. After 5 days of puromycin selection, cells were resuspended in 1× phosphate-buffered saline (PBS) and an equal amount of 2% agarose to form agarose plugs containing 1 × 10^6^ cells that were then incubated for 48 h at 50°C in lysis buffer (0.2 M EDTA [pH 8.0], 1% sodium lauryl sulfate, 1 mg/ml proteinase K). The agarose plugs were washed twice in TE buffer (10 mM Tris [pH 7.5] and 1 mM EDTA). Pulsed-field gel electrophoresis (PFGE) was performed for 23 h at 14°C with an initial switch time of 60 s and a final switch time of 120 s at 6 V/cm and an included angle of 120° as described previously (Bio-Rad CHEF Mapper) [40]. DNA was transferred to nylon membranes by established methods for Southern blotting [41]. The DNA was then detected by hybridization with α-^32^P-labeled probe specific for the EBV WP region or for cellular α-satellite repeat DNA (5’-tttcttttgatagtgcagttttgaaacattctttttaaaaaatctgcagt-3’) and visualized with a Typhoon 9410 variable-mode imager (GE Healthcare Life Sciences).

### Immunoprecipitation

Cells were extracted with lysis buffer (20 mM Tris-HCl [pH 7.4], 1 mM EDTA, 0.1 mM EGTA, 2 mM MgCl_2_, 150 mM NaCl, 1 mM Na_3_VO_4_, 1 mM NaF, 20 mM sodium glycerophosphate, 5% glycerol, 1% Triton X-100, 0.5% sodium dodecyl sulfate, 1× protease inhibitors [Sigma], 1× phosphatase inhibitors [Sigma], and 1 mM phenylmethylsulfonyl fluoride [PMSF]). After rotation for 60 min at 4°C, the lysate was centrifuged for 20 min at 16,000 × *g*, and the supernatant was recovered. The cleared extracts were used for immunoprecipitation with antibodies as indicated in the figures.

### RNA analysis

Total RNA was extracted from EBV positive cells using TRIzol (Ambion) and then further treated with DNase I (New England Biolabs). Two micrograms of total RNA were reverse transcribed using random decamers (Ambion) and Superscript IV RNase H^−^ reverse transcriptase (Invitrogen). Specific primer sets were used in real-time quantitative PCR (qPCR) assays to measure Plod1a, Plod1b, Plod2 and, Plod3 transcript levels. The values for the relative levels were calculated by ΔΔCT method.

### Flag-EBNA1 purification for proteomic mass spectrometry

293T cells were transfected with pCMV-Flag-EBNA1 OriP or Flag Vector plasmids. The cells were collected after 10 days post-transfection and washed once in 1X PBS. Cells (~10^8^) were lysed in 50 ml of Lysis buffer (50 mM Tris pH 7.5, 150 mM NaCl, 0.5% Nonidet P40, 0.5% SDS, 1 mM EDTA), 1 mM PMSF, Protease inhibitors (Catalog NO. P8340; Sigma-Aldrich) and Phosphatase inhibitors (Catalog NO. 4906837001; Roche). Lysate were spin at 16000 for 10 min and immunoprecipitated with 100 μl of Anti-Flag resin (Catalog NO. M8823; Sigma-Aldrich). Complexes were washed three times with lysis buffer containing 300 mM NaCl, 1 mM PMSF, Protease inhibitors (Catalog NO. P8340; Sigma-Aldrich) and Phosphatase inhibitors (Catalog NO. 4906837001; Roche), and eluted with Flag peptide. For EBNA 1 bound protein identification, 30 mg of Flag EBNA 1 complexes were run on a 10% precast gel (Invitrogen) for 1.5 cm and the gel was Coomassie stained. The entire stained gel regions were excised and digested with trypsin. Liquid chromatography tandem mass spectrometry (LC-MS/MS) analysis was performed using a Q Exactive HF mass spectrometer (ThermoFisher Scientific) coupled with a Nano-ACQUITY UPLC system (Waters). Samples were injected onto a UPLC Symmetry trap column (180 μm i.d. x 2 cm packed with 5 μm C18 resin; Waters), and peptides were separated by reversed phase HPLC on a BEH C18 nanocapillary analytical column (75 μm i.d. x 25 cm, 1.7 μm particle size; Waters) using a 2-h gradient formed by solvent A (0.1% formic acid in water) and solvent B (0.1% formic acid in acetonitrile). Eluted peptides were analyzed by the mass spectrometer set to repetitively scan m/z from 400 to 2000 in positive ion mode. The full MS scan was collected at 60,000 resolution followed by data-dependent MS/MS scans at 15,000 resolution on the 20 most abundant ions exceeding a minimum threshold of 20,000. Peptide match was set as preferred, exclude isotope option and charge-state screening were enabled to reject unassigned and single charged ions. Peptide sequences were identified using MaxQuant 1.5.2.8 [42]. MS/MS spectra were searched against a UniProt human protein database, EBNA1 protein sequence and a common contaminants database using full tryptic specificity with up to two missed cleavages, static carbamidomethylation of Cys, variable oxidation of Met, and variable protein N-terminal acetylation. Consensus identification lists were generated with false discovery rates set at 1% for protein and peptide identifications. Fold change was calculated using the protein intensity values.

### Mass spectrometry for post-translational modification

To identify post-translation modifications of EBNA1, Flag-EBNA 1 complexes were washed three times with buffer contains 500 mM NaCl then Flag-EBNA 1 was eluted with 3X Flag peptide and electrophoresed into an SDS-gel for a short distance. Gel regions containing Flag-EBNA 1 were digested separately with trypsin and chymotrypsin. Digests were analyzed by LC-MS/MS as described above. The MS data were searched using MaxQuant 1.6.2.3 [42]. Modifications searched were static carbamidomethylation of Cys, and variable Met oxidation, lysine hydroxylation, proline hydroxylation and protein N-terminal acetylation. Consensus identification lists were generated with false discovery rates set at 1% for protein, peptide, and site identifications.

### Cell viability assays

Cell viability was assessed 72 hours after 2’2-dipyridyl treatment using Resazurin cell proliferation/viability assay. In brief, EBV positive and negative cells were seeded onto 96-well plates and cultured overnight, followed by treatment over a ten-point concentration range of two-fold dilutions of 2’2-dipyridyl (0.39mM, 0.781mM, 1.56mM, 3.12mM, 6.25mM, 12,5 mM, 25 mM, 50 mM, 100 mM, 200 mM) (Sigma) plated in quadruplicate wells in 200 μL RPMI 1640 medium supplemented with 10% fetal bovine serum for 72 hours. As positive and negative controls, DMSO alone (0.4%) and puromycin (20 μg/ml) treated wells, respectively, were also plated in quadruplicate wells. At the end of the treatment, 20 μL of 500 mM Resazurin solution was added to each well and incubated for 6 hours at 37°C. The absorbance of each well was then detected at 590 nm under a microplate reader (CLARIOstarPlus, BMG Labtech). Cell viability was calculated as the ratio of the absorbance value to that of the control group (%) treated with 20 μg /ml puromycin.

### Cell apoptosis assay with flow cytometry

Apoptotic cells were detected using the FITC Annexin V Apoptosis Detection Kit (cat# ab14085, Abcam). EBV positive and negative cells were infected with lentivirus shCtrl or shPLOD1. After 48 hours post infection puromycin was added and selection for 3 days. The cells were then stained with Annexin V-FITC and PI according to the manufacturer’s instructions and the LSR14 Flow Cytometer (BD Biosciences). Cells were identified as viable, dead, or early or late apoptotic cells, and the percent decrease in live cell population (Q4: Annexin V(-)/PI(-) was calculated as [Q4 control-Q4 treated/Q4 control] × 100 under each experimental condition.

## Supporting information

S1 Fig RNA expression of PLODs in lymphoid cells. RT-qPCR analysis of Plod1A, Plod1B, Plod2 and Plod3 transcripts in LCLs generated with B95.8 (B) or Mutu I (M) virus, or BL lines MutuI, Kem1, and Raji. Error bars are standard deviation, n = 3 technical replicates.
(TIF)

S2 Fig PLOD1 depletion does not have strong effects on EBV lytic cycle reactivation. **A)** Raji (left) or LCL (right) transduced with lentivirus expressing shCtrl or shPLOD1 and assayed 7 days post-transduction by Western blot for PLOD1, EBNA1, ZTA, EA-D, and Actin. **B)** 293-EBV (B95-8) Bacmid containing cells were untransfected (UT), or transfected siRNA against PLOD1 (siPLOD1) or control non-targeting siRNA (siNT), and assayed by Western blot at 5 days post-transfection for PLOD1, EBNA1, ZTA, EA-D, or Actin. Quantitation via densitometry (ImageJ) of Western blot experiments (n = 3) from A. Levels of EBNA1, PLOD1, and ZTA were normalized to actin levels. Statistics were done on PRISM using paired two-tailed t-test. *p < 0.05.
(TIF)

S3 Fig Measure of shPLOD1 knock-down. **A)** RT-qPCR analysis of PLOD1 mRNA in MutuI or Raji BL cells, or B95-8 or Mutu LCLs transduced with shCtrl or shPLOD1. **B**) Western blot of cells treated as described for panel A and probed with antibody to PLOD1 (top panel) or Actin (lower panel). Each lane represents a biological replicate.
(TIF)

S4 Fig Pulse Field Gel Electrophoresis (PFGE) analysis of EBV episomes and linear forms. PFGE ladder (BioRad 0.225–2.2 Mb *S*. *cerevisiae* chromosomal DNA) shown after ethidium stain, and Southern blot of MutuI cells untreated (-) or treated with TPA/NaB for 72 hrs (+) to induce EBV lytic replication.
(TIF)

S5 Fig Mutations in K460/K461 do not disrupt EBNA1 binding to FR or DS in vivo. **A)** Western blot for FLAG-EBNA1 and Actin in HEK293T cells transfected with *oriP* plasmids expressing FLAG-EBNA1 Wt, K460A/K461A, or K460R/K461R. **B)** ChIP assays for control IgG or FLAG-EBNA1 Wt, K460AK461A, or K460R/K461R at *oriP* DS region (**left**) or FR region (**right**) for extracts shown in panel A. P-values determined by ordinary one-way ANOVA and Dunnett’s multiple comparison test. *** p<0.001, *p<0.05.
(TIF)

S6 Fig Mutations in K461 or K83A do not disrupt EBNA1 *oriP* replication or binding in vivo. **A)** Western blot for FLAG-EBNA1 and Actin in HEK293T cells transfected with *oriP* plasmids expressing FLAG-EBNA1 Wt, K461A, K460A/K461A, or K83A. **B)** Southern blot of *oriP* replication for cells shown in panel A. **C)** Quantification of *oriP* replication shown in panel B. **D-E)** ChIP assay for control IgG (**D**) or FLAG-EBNA1 (**E**) or at *oriP* DNA for EBNA1 Wt, K461A, K460A/K461A, or K83A. P-values determined by ordinary one-way ANOVA and Dunnett’s multiple comparison test. *** p<0.001, ** p < .01, *p<0.05.
(TIF)

S7 Fig PLOD1 interaction with EBNA1 is perturbed by mutations at K460 and K461. **A)** FLAG-EBNA1 WT, K460A, K461A, K460A/K461A, or K83A on *oriP*-containing plasmids were transfected into 293T cells and subject to FLAG-IP at 4 days post-transfection, followed by Western blot for PLOD1 or FLAG-EBNA1. **B)** Quantification of 3 biological replicates of experiment shown in panel A.
(TIF)

S8 Fig Mutations in EBNA1 have modest effects on protein stability. **A)** FLAG-EBNA1 WT, K460A/K461A or K460R/K461R on *oriP*-containing vectors were transfected in 293 cells for 72 hrs and then treated with 75 μg/mL of cycloheximide. Cells were lysed with 2x Laemli-SDS buffer at 0, 12, and 24 h post-cyclohexamide treatment, and then assayed by Western blot for FLAG-EBNA1. **B)** Quantification of EBNA1 protein levels for 3 replicates represented in panel B is shown in line graph below (n = 3). ** p < .01, two-tailed student t-test.
(TIF)
