# Supplementary figures and images for: Regulation of EBNA1 protein stability and DNA replication activity by PLOD1 lysine hydroxylase
Source: PLoS Pathog. 2023 Jun 1;19(6):e1010478. doi: 10.1371/journal.ppat.1010478 (PMC10263308; doi:10.1371/journal.ppat.1010478)

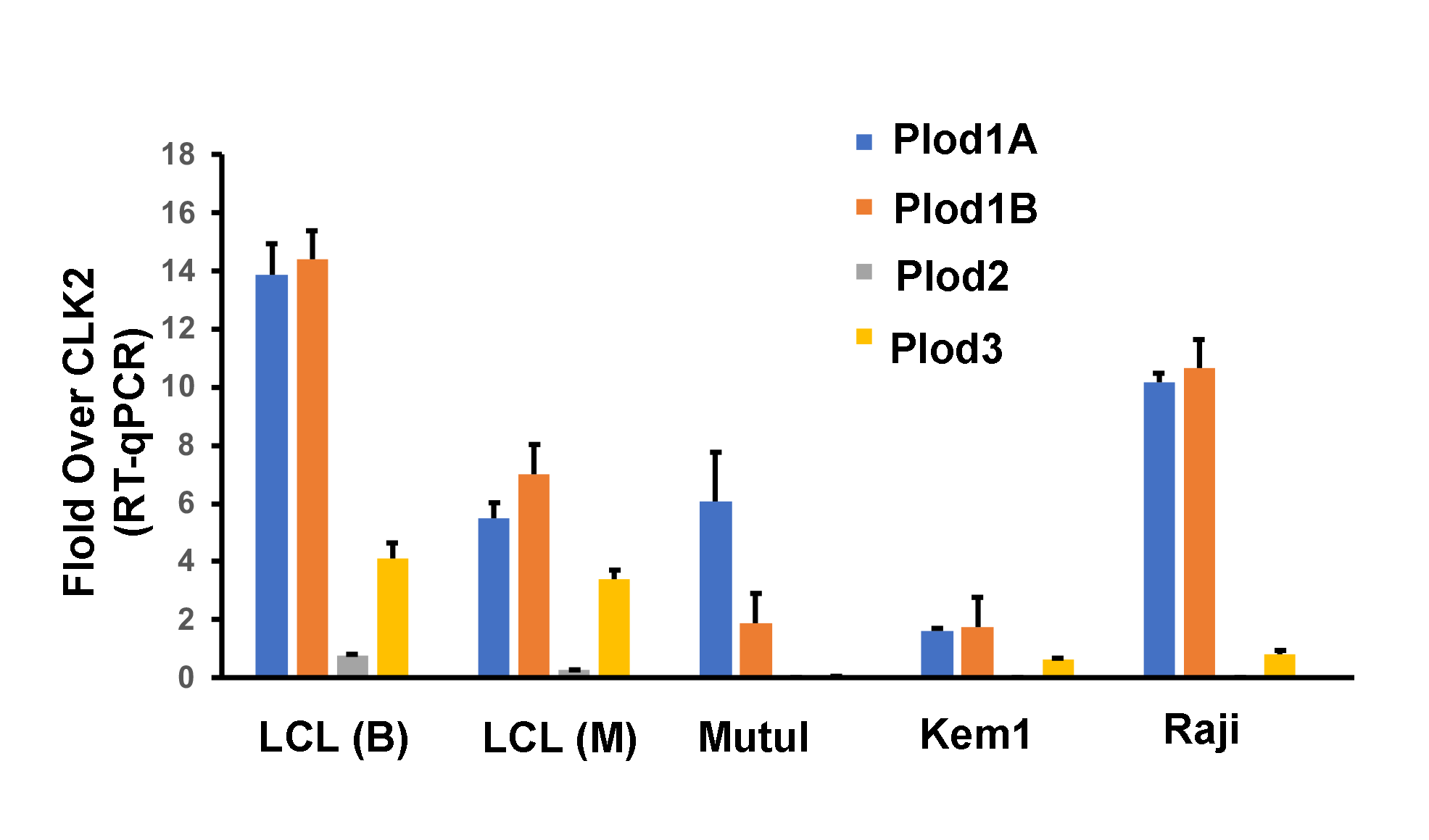

Supplement: S1 Fig — RT-qPCR analysis of Plod1A, Plod1B, Plod2 and Plod3 transcripts in LCLs generated with B95.8 (B) or Mutu I (M) virus, or BL lines MutuI, Kem1, and Raji. Error bars are standard deviation, n = 3 technical replicates. (TIF) [file ppat.1010478.s001.tif]

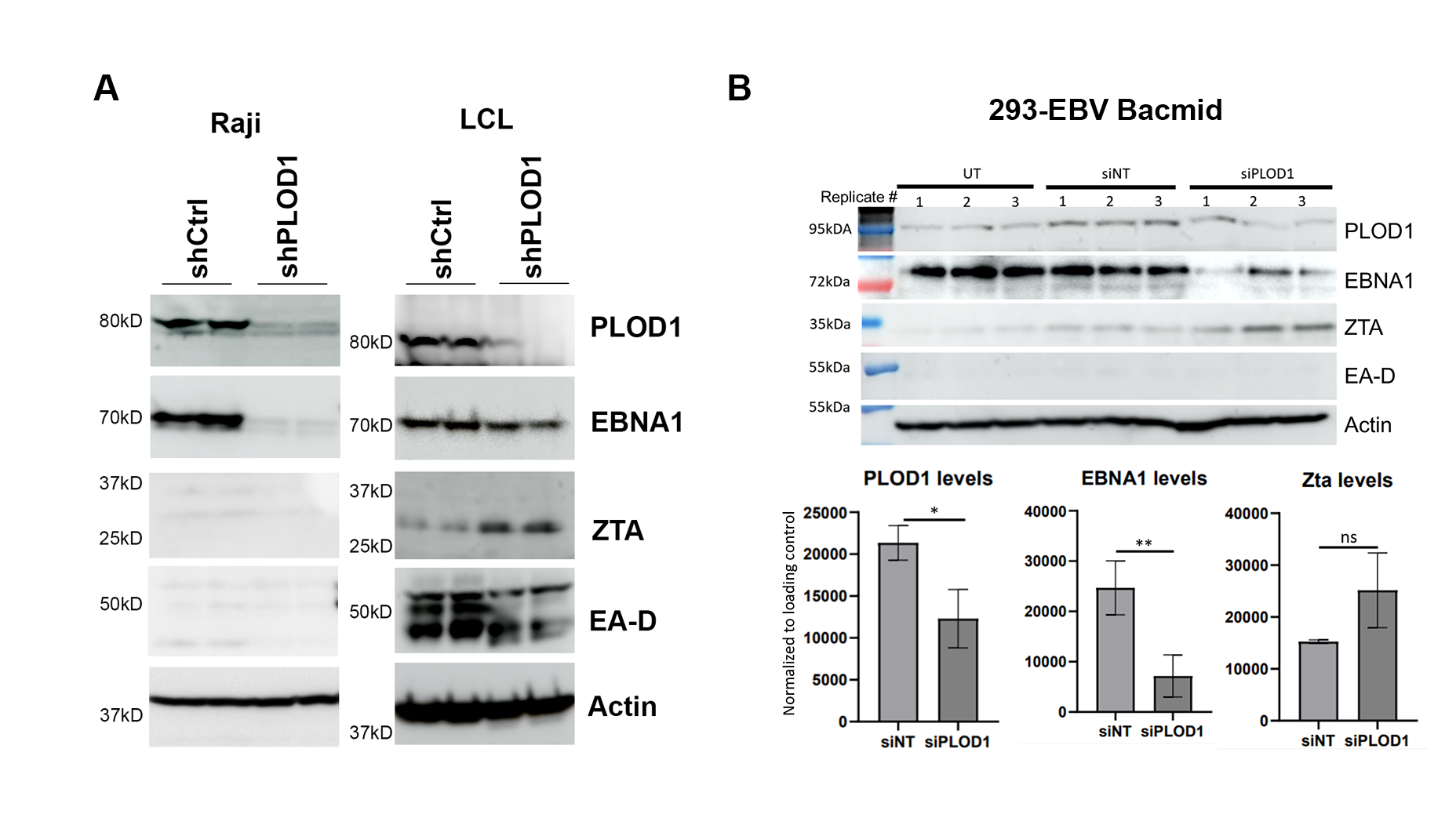

Supplement: S2 Fig — A) Raji (left) or LCL (right) transduced with lentivirus expressing shCtrl or shPLOD1 and assayed 7 days post-transduction by Western blot for PLOD1, EBNA1, ZTA, EA-D, and Actin. B) 293-EBV (B95-8) Bacmid containing cells were untransfected (UT), or transfected siRNA against PLOD1 (siPLOD1) or control non-targeting siRNA (siNT), and assayed by Western blot at 5 days post-transfection for PLOD1, EBNA1, ZTA, EA-D, or Actin. Quantitation via densitometry (ImageJ) of Western blot experiments (n = 3) from A. Levels of EBNA1, PLOD1, and ZTA were normalized to actin levels. Statistics were done on PRISM using paired two-tailed t-test. *p < 0.05. (TIF) [file ppat.1010478.s002.tif]

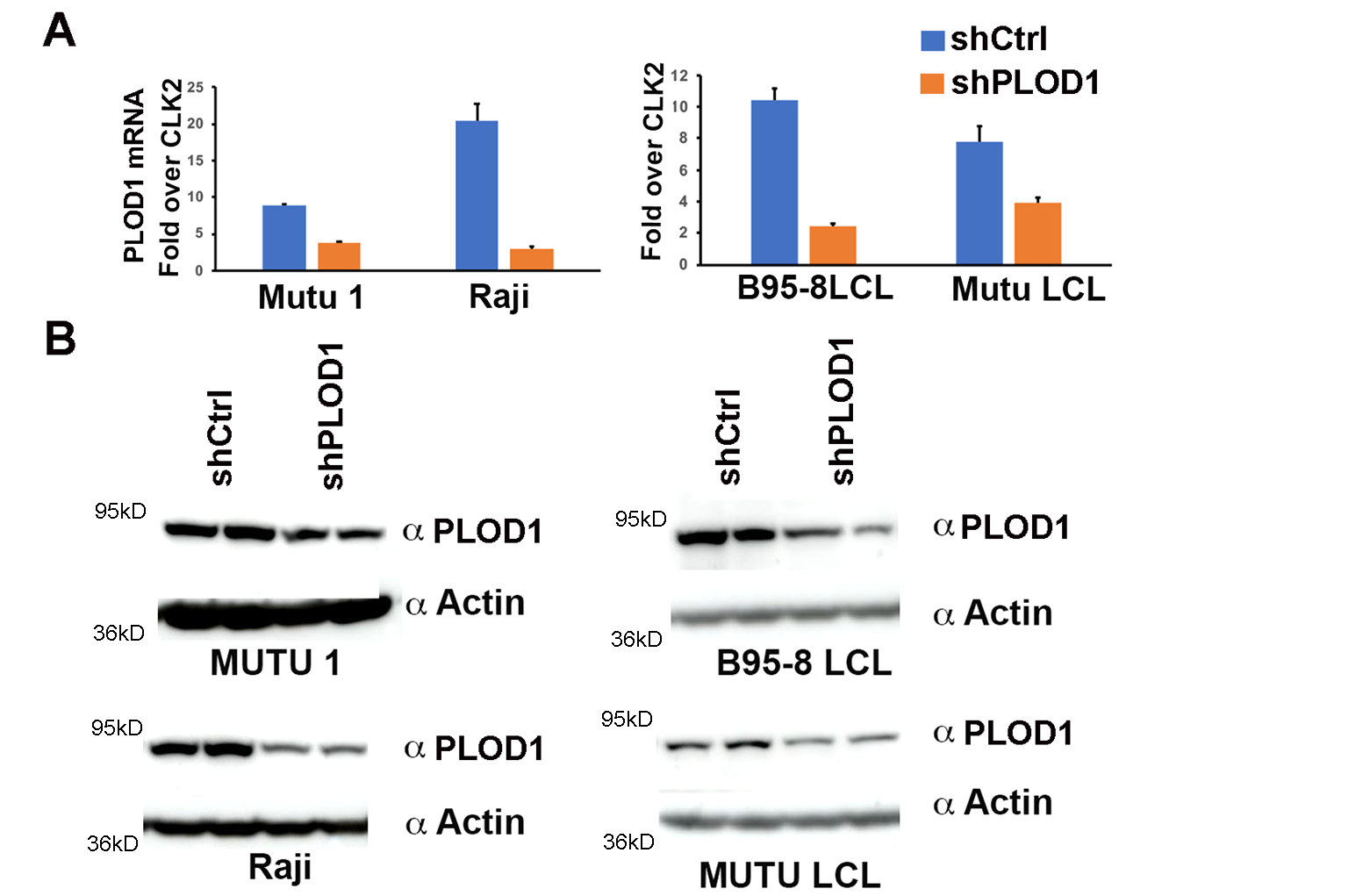

Supplement: S3 Fig — A) RT-qPCR analysis of PLOD1 mRNA in MutuI or Raji BL cells, or B95-8 or Mutu LCLs transduced with shCtrl or shPLOD1. B) Western blot of cells treated as described for panel A and probed with antibody to PLOD1 (top panel) or Actin (lower panel). Each lane represents a biological replicate. (TIF) [file ppat.1010478.s003.tif]

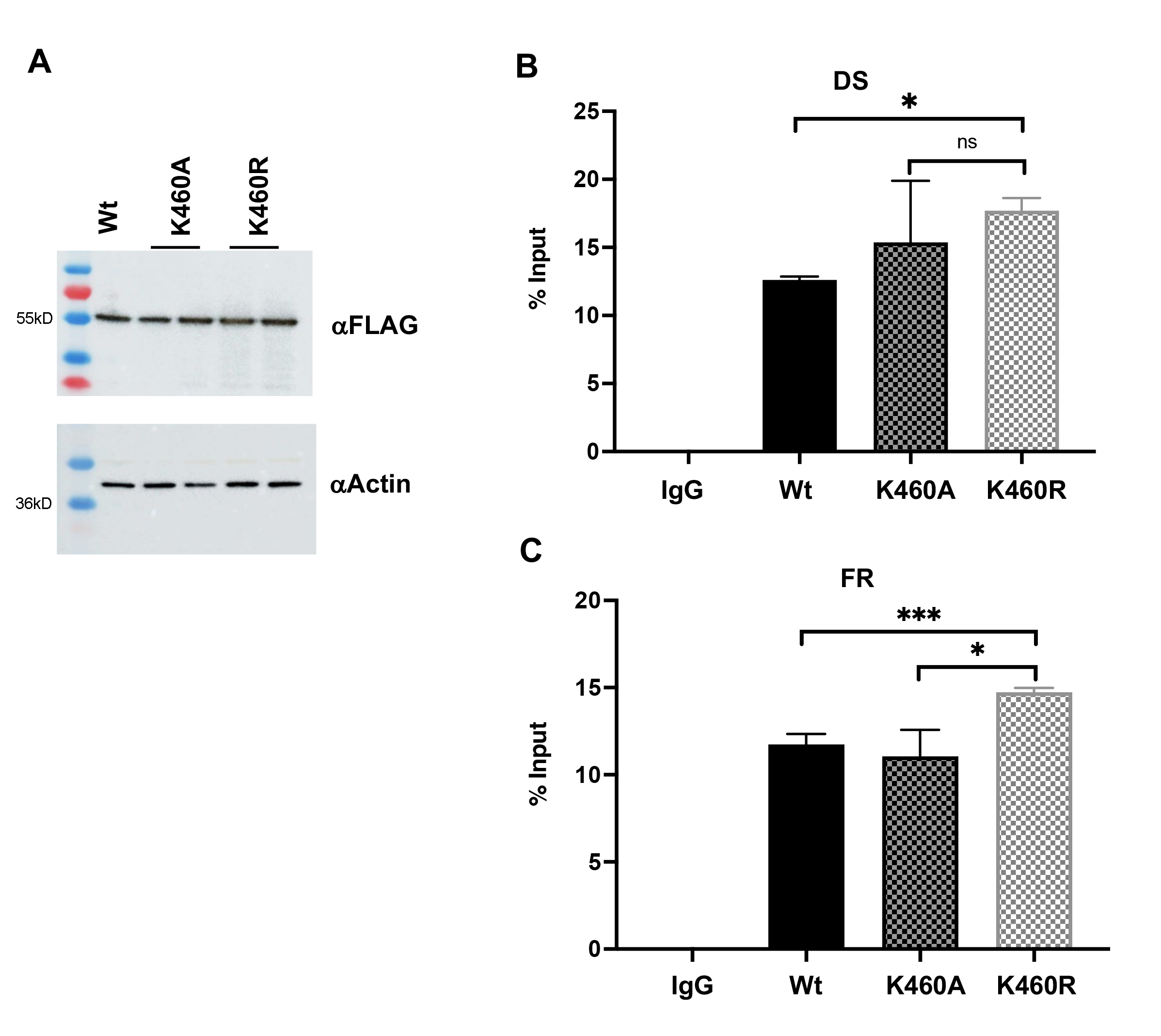

Supplement: S5 Fig — A) Western blot for FLAG-EBNA1 and Actin in HEK293T cells transfected with oriP plasmids expressing FLAG-EBNA1 Wt, K460A/K461A, or K460R/K461R. B) ChIP assays for control IgG or FLAG-EBNA1 Wt, K460AK461A, or K460R/K461R at oriP DS region (left) or FR region (right) for extracts shown in panel A. P-values determined by ordinary one-way ANOVA and Dunnett’s multiple comparison test. *** p<0.001, *p<0.05. (TIF) [file ppat.1010478.s005.tif]

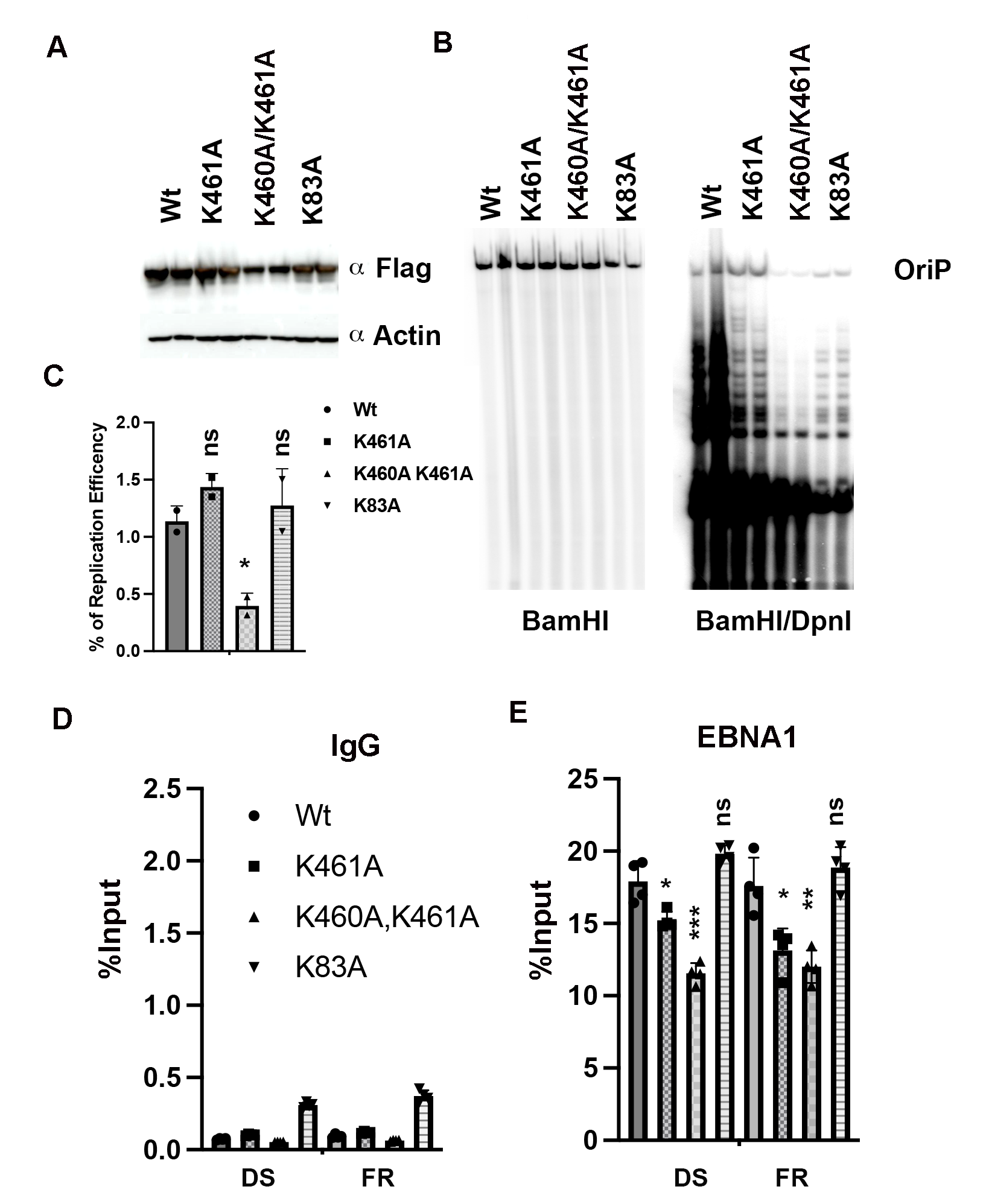

Supplement: S6 Fig — A) Western blot for FLAG-EBNA1 and Actin in HEK293T cells transfected with oriP plasmids expressing FLAG-EBNA1 Wt, K461A, K460A/K461A, or K83A. B) Southern blot of oriP replication for cells shown in panel A. C) Quantification of oriP replication shown in panel B. D-E) ChIP assay for control IgG (D) or FLAG-EBNA1 (E) or at oriP DNA for EBNA1 Wt, K461A, K460A/K461A, or K83A. P-values determined by ordinary one-way ANOVA and Dunnett’s multiple comparison test. *** p<0.001, ** p < .01, *p<0.05. (TIF) [file ppat.1010478.s006.tif]

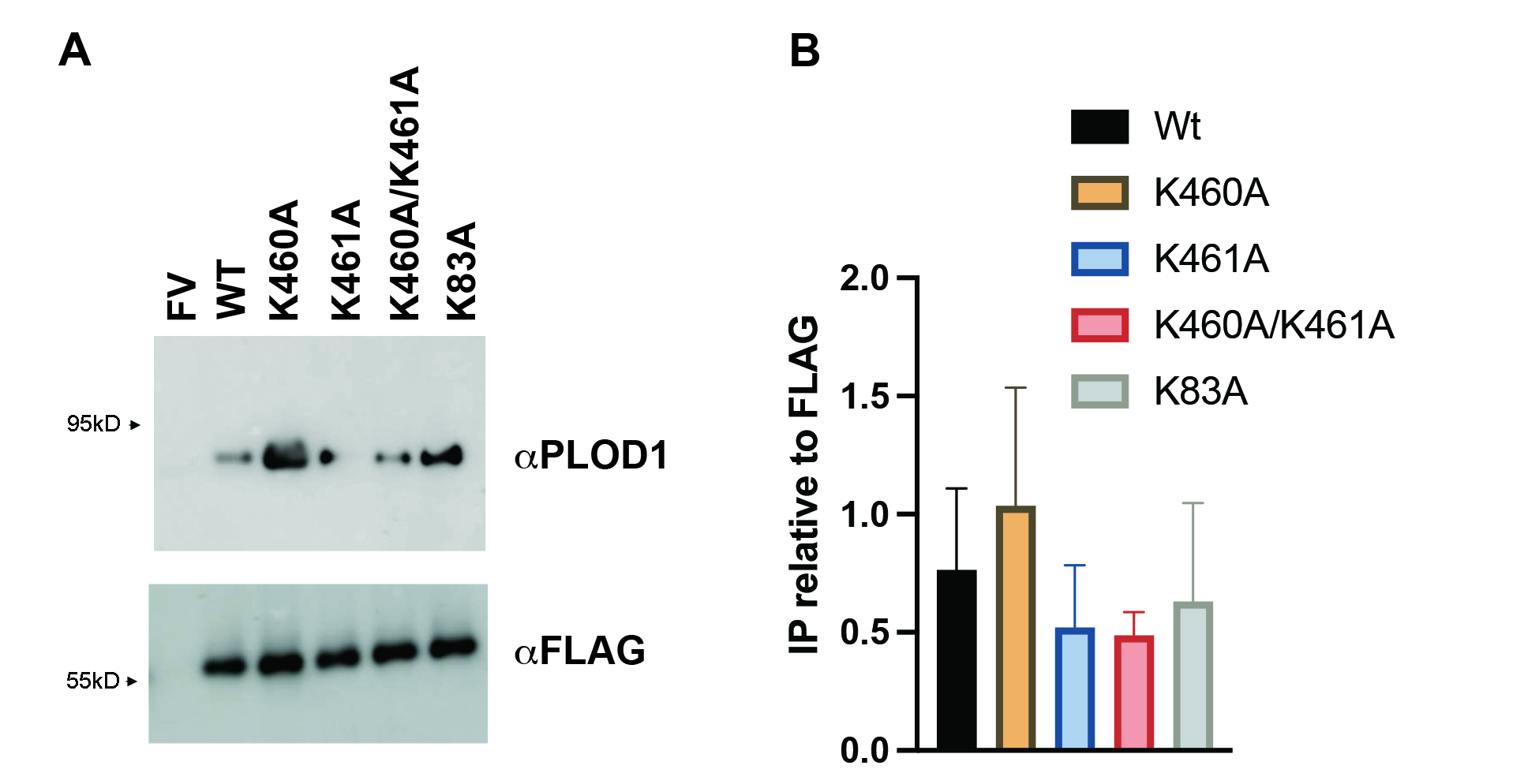

Supplement: S7 Fig — A) FLAG-EBNA1 WT, K460A, K461A, K460A/K461A, or K83A on oriP-containing plasmids were transfected into 293T cells and subject to FLAG-IP at 4 days post-transfection, followed by Western blot for PLOD1 or FLAG-EBNA1. B) Quantification of 3 biological replicates of experiment shown in panel A. (TIF) [file ppat.1010478.s007.tif]

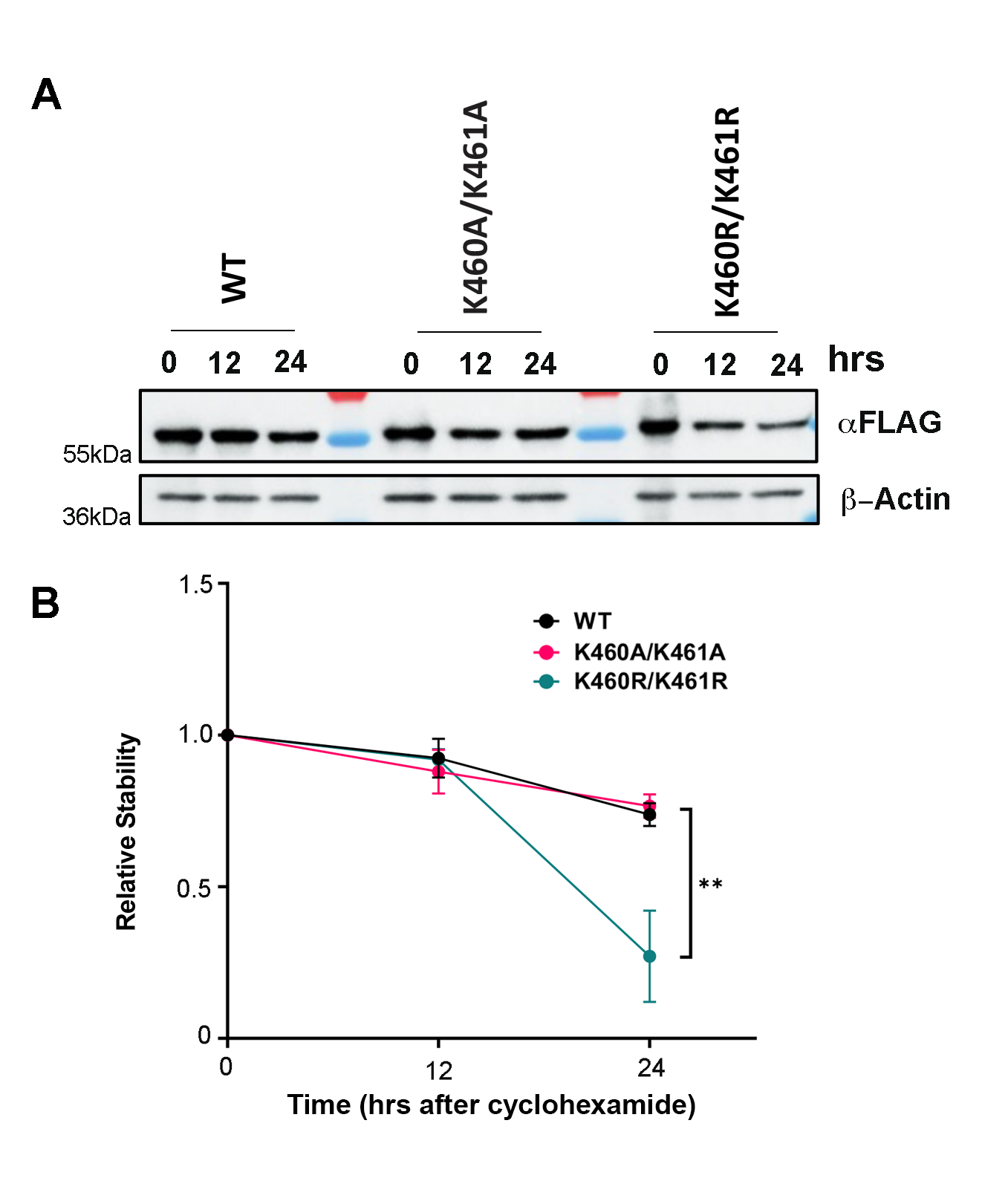

Supplement: S8 Fig — A) FLAG-EBNA1 WT, K460A/K461A or K460R/K461R on oriP-containing vectors were transfected in 293 cells for 72 hrs and then treated with 75 μg/mL of cycloheximide. Cells were lysed with 2x Laemli-SDS buffer at 0, 12, and 24 h post-cyclohexamide treatment, and then assayed by Western blot for FLAG-EBNA1. B) Quantification of EBNA1 protein levels for 3 replicates represented in panel B is shown in line graph below (n = 3). ** p < .01, two-tailed student t-test. (TIF) [file ppat.1010478.s008.tif]
